# Robotic‐Assisted Versus Laparoscopic‐Assisted Colectomy: Findings on Short‐Term Outcomes From A Multi‐Institutional Propensity Score‐Matched Study

**DOI:** 10.1002/kjm2.70163

**Published:** 2026-01-21

**Authors:** Ching‐Wen Huang, Chien‐Chih Chen, Jeng‐Fu You, Yi‐Ho Cheng, Feng‐Fan Chiang, Jaw‐Yuan Wang

**Affiliations:** ^1^ Division of Colorectal Surgery, Department of Surgery, Kaohsiung Medical University Hospital Kaohsiung Medical University Kaohsiung Taiwan; ^2^ Department of Surgery, Faculty of Medicine, College of Medicine Kaohsiung Medical University Kaohsiung Taiwan; ^3^ Department of Surgery Koo Foundation Sun Yat‐Sen Cancer Center Taipei Taiwan; ^4^ Division of Colon and Rectal Surgery, Department of Surgery Chang Gung Memorial Hospital at Linkou Tao‐Yuan Taiwan; ^5^ Department of Surgery Shin Kong Wu ho‐Su Memorial Hospital Taipei Taiwan; ^6^ Division of Colorectal Surgery, Department of Surgery Taichung Veterans General Hospital Taichung Taiwan; ^7^ Graduate Institute of Clinical Medicine, College of Medicine Kaohsiung Medical University Kaohsiung Taiwan; ^8^ Graduate Institute of Medicine, College of Medicine Kaohsiung Medical University Kaohsiung Taiwan; ^9^ Center for Cancer Research Kaohsiung Medical University Kaohsiung Taiwan

**Keywords:** laparoscopic‐assisted colectomy, multi‐institutional study, propensity score‐matching, robotic‐assisted colectomy, short‐term outcomes

## Abstract

The present study compared the short‐term outcomes and perioperative complications of robotic‐assisted colectomy (RAC) and laparoscopic‐assisted colectomy (LAC) in patients with colon cancer. This retrospective study included patients with histologically confirmed colon adenocarcinoma who underwent either RAC or LAC at five medical centers in Taiwan over the period between January 2015 and December 2021. Baseline characteristics, perioperative outcomes, and complications were obtained from operative notes and medical records. A total of 838 patients were included, with 413 undergoing RAC and 425 undergoing LAC. In the original study cohort, right colectomies and sigmoid colectomies were more frequent in the RAC group. In the matched study cohort, the median operative time was significantly longer in the RAC group (*p* = 0.0065), particularly in the subgroup undergoing left and sigmoid colectomies (*p* < 0.0001). Conversion rates were similar between the groups (*p* = 0.0616). Postoperative length of stay was significantly shorter in the RAC group by 1 day (*p* = 0.0299). Although overall postoperative complications, 30‐day mortality, and local recurrence rates were comparable between the groups (all *p* > 0.05), the patients undergoing right hemicolectomy had a significantly lower complication rate in the RAC group (*p* = 0.0018). This study is the first multi‐institutional propensity score‐matched investigation in Taiwan comparing short‐term outcomes between RAC and LAC. Our findings suggest that RAC is a safe and feasible alternative to LAC, offering comparable oncological safety with potential advantages such as a reduced length of hospital stay.

## 1 Introduction

Colorectal cancer (CRC) is a global health burden, ranking as the third most diagnosed malignancy and the second leading cause of cancer‐related mortality worldwide. In 2022, approximately 1.92 million new CRC cases were diagnosed, leading to an estimated 903,000 deaths [1]. In Taiwan, CRC has consistently been a prevalent cancer, with incidence rising from 45.5 per 100,000 in 2006 to 69.5 per 100,000 in 2021, and mortality increasing from 21.2 per 100,000 in 2010 to 29.1 per 100,000 in 2023 [2].

Surgical resection remains the primary curative treatment for localized colon cancer. Since the 1990s, laparoscopic‐assisted colectomy (LAC) has emerged as an effective minimally invasive alternative to open colectomy, offering benefits such as faster postoperative recovery, reduced analgesic use, and shorter hospital stays without compromising long‐term oncologic outcomes [3, 4, 5, 6, 7]. However, LAC's widespread adoption has been limited by technical challenges, including instrument rigidity and two‐dimensional visualization, contributing to a steep learning curve and increased procedural difficulty [8, 9].

Robotic‐assisted colectomy (RAC), introduced in the 2000s, represents a technological advancement in minimally invasive surgery, addressing many of LAC's limitations [10, 11, 12]. RAC has gained increasing traction, particularly for complex procedures demanding precise dissection in confined anatomical spaces [13]. Systems like the da Vinci robotic system (Intuitive Surgical, Sunnyvale, CA, USA) enhance surgical capabilities through high‐definition 3D visualization, tremor elimination, improved instrument articulation, and superior ergonomics. These features are especially advantageous in intricate maneuvers such as intracorporeal anastomosis during right colectomy, where RAC demonstrates superior performance over LAC [14, 15].

Despite its technical advantages, the cost‐effectiveness of RAC remains a subject of debate. High acquisition and maintenance costs, coupled with limited or no National Health Insurance coverage in Taiwan, impose a significant financial burden on patients, potentially exacerbating socioeconomic disparities in healthcare access. Consequently, there is an urgent need for real‐world evidence to evaluate the utilization and clinical value of RAC within the Taiwanese healthcare context.

This study aimed to compare the short‐term clinical outcomes of RAC and LAC in patients with colon cancer. Employing a multi‐institutional retrospective design with propensity score matching (PSM), we assessed key intraoperative and postoperative parameters, including conversion rates, lymph node yield, postoperative complications, 30‐day mortality, and readmission rates. To our knowledge, this study is the first multi‐institutional investigation in Taiwan to compare short‐term outcomes between RAC and LAC. Our findings are intended to provide crucial insights for colorectal surgeons and healthcare policymakers regarding the cost‐effectiveness and broader adoption of robotic surgery.

## 2 Materials and Methods

### 2.1 Study Population

This retrospective observational study included 838 patients who underwent either LAC or RAC between January 2015 and December 2021. The study involved five participating Taiwanese hospitals. Institutional Review Board approval was obtained from all participating centers: Kaohsiung Medical University Hospital (KMUHIRB‐E(I)‐20230267), Taichung Veterans General Hospital (CE21319A), Koo Foundation Sun Yat‐Sen Cancer Center (20220608R), Linkou Chang Gung Memorial Hospital (202102412B0), and Shin Kong Wu Ho‐Su Hospital (20220608R). All patient medical charts were reviewed up to the end of the follow‐up period to assess postoperative outcomes. Baseline characteristics and clinical outcomes were retrospectively retrieved from medical charts and operative notes. Data were collected on standardized case report forms by an investigator at each hospital and subsequently combined into a multi‐institutional dataset. Research data were stored in a password‐protected database on an external hard drive managed by the principal investigator, with access restricted to participating researchers. All investigators strictly adhered to Taiwan's Personal Data Protection Act, ensuring patient anonymity through de‐identification and pseudocode assignment. Patients diagnosed with colon cancer who underwent either LAC or RAC during the predefined index period were included. Preoperative staging for all patients included colonoscopy and computed tomography.

### 2.2 Inclusion Criteria

- Primary diagnosis of colon adenocarcinoma (ICD‐10 codes C18–C19).
- First‐time LAC or RAC using the da Vinci Si or Xi surgical system (Intuitive Surgical Inc., Sunnyvale, CA, USA) during the index period.
- Complete medical records are available.

### 2.3 Exclusion Criteria

- Age 18 years or younger at the time of surgery.
- Underwent emergent surgeries.
- Lost to follow‐up after surgery.
- Presence of multiple cancers or metastasis.
- History of multiple abdominal surgeries: an operative history requiring laparotomy or laparoscopy (i.e., not minor non‐abdominal procedures).

### 2.4 Outcomes

The following clinicopathological features and perioperative parameters were evaluated: age, sex, TNM (tumor, node, and metastasis) classification (based on AJCC and UICC criteria) [16] tumor location, body mass index (BMI), American Society of Anesthesiologists (ASA) score, Charlson Comorbidity Index (CCI) score, and preoperative neoadjuvant therapy status.

Intraoperative safety measures included death, surgical procedures performed, conversion to open surgery, operation time (defined as total duration from skin incision to wound closure), estimated blood loss, and number of blood transfusions.

Postoperative clinical outcomes were analyzed during predischarge and postdischarge periods, encompassing length of hospital stay, rehospitalization within 30 days postoperative, reoperation within 30 days postoperative, and death within 30 days postoperative.

### 2.5 Study Period

Patients who underwent LAC or RAC between January 1, 2015, and December 31, 2021, were included. The index date was the date of the first admission for initial colon resection. A 6‐month preoperative history was reviewed for baseline health status. All medical charts were examined until the end of the follow‐up period (January 31, 2022) for postoperative outcomes.

Key terms were defined as:

- *Index Period*: January 1, 2015, to December 31, 2021.
- *Index Surgery*: The initial colectomy performed.
- *Index Hospitalization*: The first hospitalization for colectomy during the index period.
- *Pre‐disease (Treatment) History*: The 6‐month baseline period prior to the index date.
- *Follow‐Up Period*: The 30‐day period following hospital discharge.

### 2.6 Data Management

#### 2.6.1 Confidentiality and Quality Control

Strict confidentiality was maintained. Patient data were de‐identified before analysis, with pseudocodes assigned. Due to the retrospective design, informed consent was not obtained. Data were stored in a password‐protected database on an external hard drive, accessible only to researchers and supervised by the principal investigator. Data accuracy was ensured through review by the principal investigator and lead researcher at each hospital.

#### 2.6.2 Case Report Form

The study's case report form was the primary data collection instrument. All required data were recorded, with explanations for any missing entries. “NA” was used for unavailable items. Entries were locked after initial completion, and any corrections were reviewed collaboratively with the initial investigator, with all changes documented systematically.

#### 2.6.3 Record Retention

All source data and documents were securely retained by the principal investigator in a designated storage facility, accessible to authorized authorities. Upon study completion, all documents were encrypted and password‐protected.

### 2.7 Study Monitoring and Ethical Considerations

#### 2.7.1 Monitoring and Inspection

Principal investigators at each hospital ensured adequate time for monitoring activities and guaranteed access to all study‐related documents for monitors or quality assurance reviewers. Participation implied acceptance of potential inspections by government regulatory authorities and hospital compliance offices.

#### 2.7.2 Ethical Considerations

This study adhered to Taiwanese government regulations and research policies of participating institutions. The principal investigator was responsible for informing IRBs and research groups of any protocol amendments.

### 2.8 Statistical Analysis

Baseline patient characteristics and outcome variables were summarized using descriptive statistics for both surgical groups. Continuous variables were reported as medians with interquartile ranges (IQR), and categorical variables as frequencies (n) and percentages (%). Subgroup analyses were performed based on procedure type and surgical technique. All statistical analyses were conducted using R software (version 4.2.1; Free Software Foundation Inc., Vienna, Austria).

To mitigate selection bias inherent in observational studies, propensity score matching (PSM) was employed. PSM balanced the LAC and RAC groups based on sex, age, ASA classification, cancer stage, CCI score, and type of surgery (right, left, or sigmoid colectomy). A nearest‐neighbor algorithm with a 1:1 ratio and a caliper width of 0.1 was used. Covariate balance was assessed post‐matching.

Initial statistical comparisons of baseline characteristics utilized Student's *t*‐test for normally distributed continuous variables and the chi‐square test for categorical variables. The Mann–Whitney U test was applied for non‐normally distributed continuous variables. If significant imbalances persisted after initial PSM, propensity scores were re‐estimated via logistic regression, and a refined matching process was conducted using a narrower caliper width of 0.02. Outcomes were compared between matched and unmatched populations. For the matched cohort, paired *t*‐test or Wilcoxon signed‐rank test was used for continuous variables, and the McNemar test for categorical variables. Subgroup analyses were conducted when clinically relevant or statistically justified.

## 3 Results

### 3.1 Patient Characteristics and Perioperative Outcomes

Between January 2015 and December 2021, 838 patients with colon cancer undergoing minimally invasive colon resection at six Taiwanese hospitals were evaluated. The demographic and baseline characteristics of the patients before and after PSM are presented in Table 1.

**TABLE 1:** Demographic and baseline characteristics of the 838 patients.

Characteristic	Overall			After matching		
	RAC (*N* = 413)	LAC (*N* = 425)	*p*	RAC (*N* = 334)	LAC (*N* = 334)	*p*
Age (years, median) (IQR)	62 (17)	63 (15)		61 (16)	63 (15)	
18~70	308 (74.6%)	319 (75.1%)	0.399	267 (79.9%)	255 (76.4%)	0.050
> 70	105 (25.4%)	106 (24.9%)		67 (20.1%)	79 (23.6%)	
Sex						
Male	211 (51.1%)	229 (53.9%)	0.459	194 (58.1%)	186 (56.7%)	0.585
Female	202 (48.9%)	196 (46.1%)		140 (41.9%)	148 (44.3%)	
BMI (kg/m^2^)						
Normal (< 24)	185 (44.8%)	189 (44.5%)	0.980	146 (43.7%)	155 (46.4%)	0.534
Overweight (≥ 24)	228 (55.2%)	236 (55.5%)		188 (56.3%)	179 (53.6%)	
Surgery type						
Right colectomy	136 (32.9%)	124 (29.2%)	0.008^a^	119 (35.6%)	105 (31.4%)	0.506
Left colectomy	44 (10.7%)	77 (18.1%)		39 (11.7%)	39 (11.7%)	
Sigmoid Colectomy	233 (56.4%)	224 (52.7%)		176 (52.7%)	190 (56.9%)	
CCI scores						
0–1	376 (91.1%)	379 (89.2%)	0.615	306 (91.6%)	304 (91.0%)	0.956
2	22 (5.3%)	26 (6.1%)		16 (4.8%)	17 (5.1%)	
3	15 (3.6%)	20 (4.7%)		12 (3.6%)	13 (3.9%)	
ASA score						
1	24 (5.8%)	16 (3.8%)	0.123	19 (5.7%)	14 (4.2%)	0.668
2	218 (52.8%)	267 (62.8%)		191 (57.2%)	197 (59.0%)	
3	169 (41.0%)	142 (33.4%)		124 (37.1%)	123 (36.8%)	
4	1 (0.2%)	0 (0.0%)		0 (0.0%)	0 (0.0%)	
NA	1 (0.2%)	0 (0.0%)		0 (0.0%)	0 (0.0%)	
AJCC stage						
I	86 (20.8%)	116 (27.3%)	0.002^a^	79 (23.7%)	70 (21.0%)	0.605
II	129 (31.2%)	111 (26.1%)		99 (29.6%)	96 (28.7%)	
III	196 (47.5%)	184 (43.3%)		156 (46.7%)	168 (50.3%)	
NA	2 (0.5%)	14 (3.3%)		0 (0.0%)	0 (0.0%)	
Tumor size (cm, median) (IQR)	3.3 (2.7)	3.2 (2.5)	0.762	3.2 (2.52)	3.5 (3)	0.197

Abbreviations: AJCC, American Joint Commission on Cancer; ASA, American Society of Anesthesiologists; BMI, body mass index; CCI, Charlson Comorbidity Index; IQR, interquartile range; LAC, laparoscopic‐assisted colectomy; NA, not available; RAC, robotic‐assisted colectomy.

^a^ *p* < 0.05.

Before PSM, 413 patients underwent RAC and 425 underwent LAC. No significant differences were observed between the groups in age, sex, BMI, ASA score, CCI score, or tumor size. However, the distribution of surgical procedures (*p* = 0.0083) and cancer stages (*p* = 0.0018) differed significantly. The RAC group had a lower percentage of left colectomies and more right hemicolectomies and sigmoidectomies. Also, the RAC group had a higher percentage of stage II and III cancers and fewer stage I cancers.

After PSM, 668 cases were included (334 RAC; 334 LAC). Baseline characteristics were well balanced, with no significant differences identified (all *p* > 0.05). Separate subgroup analyses were conducted for right colectomy and combined left/sigmoid colectomy cohorts, both demonstrating well‐balanced baseline characteristics after PSM (Tables 2 and 3).

**TABLE 2:** Demographic and baseline characteristics of patients undergoing right colectomy.

Characteristic	Overall			After matching		
	RAC (*N* = 136)	LAC (*N* = 124)	*p*	RAC (*N* = 103)	LAC (*N* = 103)	*p*
Age (years, median) (IQR)	66 (14)	66 (14)		62 (20.5)	65 (13)	
18~70	96 (75.6%)	83 (66.9%)	0.082	73 (70.9%)	73 (70.9%)	0.132
> 70	40 (29.4%)	41 (33.1%)		30 (29.1%)	30 (29.1%)	
Sex						
Male	66 (48.5%)	58 (46.8%)	0.874	48 (46.6%)	48 (46.6%)	> 0.99
Female	70 (51.5%)	66 (53.2%)		55 (53.4%)	55 (53.4%)	
BMI (kg/m^2^)						
Normal (< 24)	63 (53.7%)	62 (50.0%)	0.639	47 (45.6%)	52 (50.5%)	0.577
Overweight (≥ 24)	73 (46.3%)	62 (50.0%)		56 (54.4%)	51 (49.5%)	
CCI scores						
0–1	125 (91.9%)	106 (85.5%)	0.188	100 (97.1%)	100 (97.1%)	> 0.99
2	6 (4.4%)	7 (5.6%)		1 (1.0%)	1 (1.0%)	
3	5 (3.7%)	11 (8.9%)		2 (1.9%)	2 (1.9%)	
ASA score						
1	11 (8.1%)	7 (5.7%)	0.363	8 (7.8%)	6 (5.8%)	0.262
2	61 (44.8%)	66 (53.2%)		49 (47.6%)	61 (59.2)	
3	64 (47.1%)	51 (41.1%)		46 (44.6%)	36 (35.0%)	
4	0 (0.0%)	0 (0.0%)		0 (0.0%)	0 (0.0%)	
AJCC stage						
I	32 (23.5%)	116 (27.3%)	0.083	18 (17.5%)	25 (24.3%)	0.383
II	51 (37.5%)	111 (26.1%)		40 (38.8%)	32 (31.1%)	
III	53 (39.0%)	184 (43.3%)		45 (43.7%)	46 (46.6%)	
NA	0 (0.0%)	14 (3.3%)		0 (0.0%)	0 (0.0%)	
Tumor size (cm, median) (IQR)	3.5 (2.6)	3.3 (3.0)	0.979	3.5 (2.3)	3.2 (2.6)	0.259

Abbreviations: AJCC, American Joint Commission on Cancer; ASA, American Society of Anesthesiologists; BMI, body mass index; CCI, Charlson Comorbidity Index; IQR, interquartile range; LAC, laparoscopic‐assisted colectomy; NA, not available; RAC, robotic‐assisted colectomy.

**TABLE 3:** Demographic and baseline characteristics of patients undergoing left and sigmoid colectomy.

Characteristic	Overall			After matching		
	RAC (*N* = 277)	LAC (*N* = 301)	*p*	RAC (*N* = 239)	LAC (*N* = 239)	*p*
Age (years, median) (IQR)	62 (16)	62 (15)		61 (16)	63 (15)	
18~70	212 (76.5%)	236 (78.4%)	0.977	267 (79.9)	255 (76.4)	0.050
> 70	65 (23.5%)	65 (21.6%)		67 (20.1)	79 (23.6)	
Sex						
Male	211 (51.1)	229 (53.9)	0.321	194 (58.1)	186 (56.7)	0.585
Female	202 (48.9)	196 (46.1)		140 (41.9)	148 (44.3)	
BMI (kg/m^2^)						
Normal (< 24)	185 (44.8)	189 (44.5)	0.715	146 (43.7)	155 (46.4)	0.534
Overweight (≥ 24)	228 (55.2)	236 (55.5)		188 (56.3)	179 (53.6)	
CCI scores						
0–1	376 (91.1)	379 (89.2)	0.888	306 (91.6)	304 (91.0)	0.956
2	22 (5.3)	26 (6.1)		16 (4.8)	17 (5.1)	
3	15 (3.6)	20 (4.7)		12 (3.6)	13 (3.9)	
ASA score						
1	24 (5.8)	16 (3.8)	0.045^a^	19 (5.7)	14 (4.2)	0.668
2	218 (52.8)	267 (62.8)		191 (57.2)	197 (59.0)	
3	169 (41.0)	142 (33.4)		124 (37.1)	123 (36.8)	
4	1 (0.2)	0 (0.0)		0 (0.0)	0 (0.0)	
NA	1 (0.2)	0 (0.0)		0 (0.0)	0 (0.0)	
AJCC stage						
I	86 (20.8)	116 (27.3)	0.004^a^	79 (23.7)	70 (21.0)	0.605
II	129 (31.2)	111 (26.1)		99 (29.6)	96 (28.7)	
III	196 (47.5)	184 (43.3)		156 (46.7)	168 (50.3)	
NA	2 (0.5)	14 (3.3)		0 (0.0)	0 (0.0)	
Tumor size (cm, median) (IQR)	3.3 (2.7)	3.2 (2.5)	0.526	3.2 (2.52)	3.5 (3)	0.197

Abbreviations: AJCC, American Joint Commission on Cancer; ASA, American Society of Anesthesiologists; BMI, body mass index; CCI, Charlson Comorbidity Index; IQR, interquartile range; LAC, laparoscopic‐assisted colectomy; NA, not available; RAC, robotic‐assisted colectomy.

^a^ *p* < 0.05.

### 3.2 Intraoperative Safety and Clinical Outcomes After PSM

The intraoperative safety and clinical outcomes after PSM of the patients are summarized in Table 4. The median operative time was significantly longer in the RAC group (260 vs. 225 min, *p* = 0.0065) in the overall cohort. However, this difference was driven solely by the combined left and sigmoid colectomy group (285 vs. 220 min, *p* < 0.0001). For right colectomy, operative time was comparable (188.5 vs. 178.5 min, *p* = 0.1468).

**TABLE 4:** Intraoperative safety and clinical outcomes after propensity score matching.

Characteristic	Overall			Sub‐procedure analysis					
				Right colectomy			Combined left and sigmoid colectomy		
	RAC (*N* = 334)	LAC (*N* = 334)	*p*	RAC (*N* = 103)	LAC (*N* = 103)	*p*	RAC (*N* = 239)	LAC (*N* = 239)	*p*
Operation time (min, median) (IQR)	260 (155)	225 (160)	0.006^a^	230 (188.5)	199 (178.5)	0.147	285 (136.75)	220 (148.75)	< 0.001^a^
Conversion	0 (0.0%)	5 (1.5%)	0.062	0 (0.0%)	2 (1.9%)	0.498	0 (0.0%)	1 (0.4%)	> 0.99
Blood Loss (mL, IQR)	30 (20)	30 (20)	0.738	30 (20)	30 (20)	0.438	30 (20)	30 (25)	0.433
Transfusion	6 (1.8%)	2 (0.6%)	0.286	4 (3.9%)	1 (1.0%)	0.369	1 (0.4%)	0 (0.0%)	> 0.99
Median harvested lymph node (IQR)	20 (13)	19 (10)	0.068	24 (13.75)	22 (8)	0.0056^a^	19 (12)	18 (9)	0.717
Positive surgical margin	8 (2.4%)	4 (1.2%)	0.383	0 (0.0%)	0 (0.0%)	NA	0 (0.0%)	0 (0.0%)	NA

Abbreviations: IQR, interquartile range; LAC, laparoscopic‐assisted colectomy; NA, not available; RAC, robotic‐assisted colectomy.

^a^ *p* < 0.05.

No statistically significant differences were observed between the two groups in conversion rate, estimated blood loss, blood transfusion, harvested lymph nodes, or positive surgical margin. Notably, no RAC patients required conversion to open surgery, compared to five LAC patients (1.5%) (*p* = 0.0616).

### 3.3 Postoperative Safety and Clinical Outcomes After PSM

The postoperative safety and clinical outcomes of the patients after PSM are summarized in Table 5. In the overall cohort, RAC patients had a significantly shorter median postoperative hospital stay (1 day shorter, *p* = 0.0299). This difference was primarily observed in the combined left and sigmoid colectomy subgroup (*p* = 0.0262), but not in the right colectomy group (*p* = 0.2574).

**TABLE 5:** Postoperative safety and clinical and oncological outcomes after propensity score matching.

Characteristic	Overall			Sub‐procedure analysis					
				Right colectomy			Combined left and sigmoid colectomy		
	RAC (*N* = 334)	LAC (*N* = 334)	*p*	RAC (*N* = 103)	LAC (*N* = 103)	*p*	RAC (*N* = 239)	LAC (*N* = 239)	*p*
Postoperative LOS (days)	7 (2)	8 (2)	0.030^a^	8 (3)	8 (3.5)	0.257	7 (2)	8 (3)	0.026^**a**^
Readmission	6 (1.8%)	6 (1.8%)	> 0.99	0 (0.0%)	3 (2.9%)	0.246	4 (1.7%)	4 (1.7%)	> 0.99
Reoperation	5 (1.5%)	10 (3.0%)	0.286	1 (1.0%)	5 (4.9%)	0.059	3 (1.3%)	8 (3.4%)	0.221
Complications	32 (9.6%)	46 (13.8%)	0.117	6 (5.8%)	26 (25.2%)	0.002^a^	20 (8.4%)	24 (10.0%)	0.636
Anastomosis leakage	8 (2.4%)	10 (3.0%)	0.811	1 (1.0%)	5 (4.9%)	0.212	3 (1.3%)	6 (2.6%)	0.5034
Surgical site infection	8 (2.4%)	7 (2.1%)	> 0.99	1 (1.0%)	3 (2.9%)	0.621	6 (2.6%)	5 (2.1%)	> 0.99
Ileus	13 (3.9%)	19 (5.7%)	0.365	5 (4.9%)	12 (11.7%)	0.128	8 (3.4%)	8 (3.4%)	> 0.99
Internal bleeding	2 (0.6%)	3 (0.9%)	> 0.99	0 (0.0%)	2 (1.9%)	0.498	1 (0.4%)	1 (0.4%)	> 0.99
Urinary retention	1 (0.3%)	3 (0.9%)	0.624	0 (0.0%)	0 (0.0%)	NA	0 (0.0%)	4 (1.7%)	0.123
Urinary tract infection	1 (0.3%)	1 (0.3%)	> 0.99	0 (0.0%)	0 (0.0%)	NA	1 (0.4%)	1 (0.4%)	> 0.99
Pneumonia	0 (0.0%)	4 (1.2%)	0.1249	0 (0.0%)	3 (2.9%)	0.2463	2 (0.8%)	1 (0.4%)	> 0.99
Intraabdominal abscess	2 (0.6%)	5 (0.8%)	0.451	0 (0.0%)	2 (1.9%)	0.498	1 (0.4%)	4 (1.7%)	0.372
Sepsis	2 (0.6%)	5 (1.5%)	0.451	0 (0.0%)	3 (2.9%)	0.246	2 (0.8%)	2 (0.8%)	> 0.99
30‐day mortality	1 (0.3%)	1 (0.3%)	> 0.99	1 (1.0%)	1 (0.3%)	> 0.99	0 (0.0%)	0 (0.0%)	NA
Local recurrence	8 (2.4%)	8 (2.4%)	> 0.99	3 (2.9%)	3 (2.9%)	> 0.99	5 (2.1%)	5 (2.1%)	> 0.99

Abbreviations: LAC, laparoscopic‐assisted colectomy; LOS, length of hospital stay; NA, not available; RAC, robotic‐assisted colectomy.

^**a**^ *P* < 0.05.

Postoperative complication rates and 30‐day mortality were comparable between groups in the overall analysis (*p* = 0.1168 and *p* = 1.0000, respectively). However, in the right hemicolectomy subgroup, the RAC group showed a significantly lower complication rate than the LAC group (*p* = 0.0018). This advantage was not seen in the left or sigmoid colectomy subgroups (*p* = 0.1168 and *p* = 0.6355, respectively).

Regarding oncologic safety, local recurrence rates were not significantly different between the two approaches in the overall analysis or in subgroup analyses (*p* = 1.0000 for all).

## 4 Discussion

Since its introduction nearly two decades ago, robot‐assisted surgery has been increasingly used in colorectal procedures. Studies have demonstrated that robotic‐assisted resection for rectal cancer is a feasible and safe technique, with outcomes comparable to those of laparoscopic surgery [17, 18]. Robotic‐assisted rectal resection may offer several benefits, such as lower conversion rates, faster recovery of bowel function, shorter hospital stays [19, 20], and advantages in operating on patients with anatomically challenging pelvises.

However, the advantages of robotic‐assisted surgery over laparoscopic surgery in colon resection remain controversial and less extensively studied, particularly concerning oncological outcomes. This study is the first multi‐institutional comparison of robotic and laparoscopic approaches for colon cancer in Taiwan, utilizing a comprehensive dataset of over 800 Taiwanese patients. Unlike most studies focusing on single colectomy types, our analysis includes outcomes for right colectomy, combined left and sigmoid colectomy, and an overall analysis, providing broad insights.

After PSM, no significant differences were found in intraoperative outcomes (conversion rate, intraoperative transfusion, blood loss) between RAC and LAC in the overall or combined left/sigmoid colectomy analyses. The conversion rate to open surgery in our study was 0% for RAC and 1.5% for LAC, consistent with reported ranges for RAC (0%–6.67%) and LAC (0%–10.44%) [21]. While meta‐analyses on right colectomy have reported better conversion rates, length of hospital stay, time to first flatus, rate of overall complications, and reduced blood loss for RAC [22, 23], our analysis of right colectomy (with 103 matched patients per group) did not reproduce these findings, suggesting comparable outcomes in our cohort. This discrepancy might be attributed to various factors, including the specific surgical techniques employed across different institutions, the varying experience levels of surgeons with both platforms, or subtle differences in patient populations that were not fully captured by our PSM model. Further prospective studies with standardized protocols are needed to elucidate these potential influences.

RAC is conventionally associated with longer operative times than LAC, primarily due to docking and setup [21, 22, 23, 24, 25]. Our study observed a similar trend, with RAC having a median operative time approximately 35 min longer than LAC overall. This pattern was consistent across subgroups, though the difference for right colectomy was non‐significant. This may reflect early implementation experience, including unfamiliarity with trocar placement, robot setup time, and coordination. However, as surgical teams gain more experience and efficiency with robotic platforms, it is anticipated that operative times for RAC will progressively decrease, potentially narrowing or even eliminating this time difference compared to LAC. This trend has been observed in other surgical specialties as robotic surgery matures.

The rate of positive surgical margins was comparable between groups. Notably, in the right colectomy cohort, significantly more lymph nodes were harvested in the RAC group than in the LAC group, with the difference reaching significance [22]. This aligns with meta‐analyses suggesting RAC leads to higher lymph node yields in right colectomy. The robotic platform's enhanced visualization, instrument dexterity, and ergonomics may facilitate precise dissection, crucial for D3 lymph node dissection. The superior 3D vision and wristed instruments of the robotic system allow for meticulous dissection around vascular structures and lymph node basins, potentially leading to a more thorough oncological clearance [17]. While our retrospective study could not definitively determine whether the increased lymph node count in the RAC group was attributable to a higher rate of D3 dissections due to missing procedural details, the finding itself suggests a potential oncological advantage that warrants further investigation in future prospective studies with detailed procedural documentation.

Regarding postoperative outcomes, our overall analysis showed no significant differences in complications between RAC (4.8%) and LAC (6.9%). However, in the right colectomy subgroup, RAC exhibited a significantly lower overall postoperative complication rate (*p* = 0.0018), with apparently lower incidences of anastomosis leakage, ileus, sepsis, and bleeding, though these individual differences were not significant. This finding contrasts with some meta‐analyses showing no significant differences in overall postoperative outcomes for right colectomy [22], but aligns with others reporting fewer complications for RAC [21]. For left colectomy, Solaini et al. demonstrated lower overall complication rates for RAC, a trend not observed in our left/sigmoid colectomy subgroups [12]. This observed benefit in right colectomy could be attributed to the robotic system's ability to facilitate intracorporeal anastomosis with greater precision and less tissue manipulation, which is often a more complex maneuver in right‐sided resections. The enhanced visualization and stability offered by the robot may contribute to a more secure anastomosis and reduced rates of related complications.

Anastomotic leakage, a major cause of morbidity, occurred at a similar rate (1.35%) in both groups in our matched cohort, consistent with other studies [12, 26]. Readmission and reoperation rates were also similar. However, the postoperative length of hospital stay was significantly shorter in the RAC group in both the overall cohort and the combined left and sigmoid colectomy subgroup. This difference was not significant in the right colectomy cohort, possibly due to smaller sample size, though two meta‐analyses have reported significantly shorter hospital stays for robotic‐assisted right colectomy [23, 27]. The reduction in hospital stay for RAC patients, particularly in left and sigmoid colectomies, suggests a faster overall recovery, which could translate to significant benefits for both patients and healthcare systems, including reduced healthcare costs and earlier return to normal activities. This finding, even with the non‐significant difference in right colectomy, highlights a tangible patient‐centered benefit of robotic surgery.

The present study has several limitations. First, its retrospective design inherently limits causal inference. The inherent limitations of a retrospective design will introduce selection and reporting biases and the inability to establish definitive causality, even with the use of PSM. PSM helps mitigate selection bias, but cannot eliminate all confounding factors. Second, surgical technique variations among surgeons across multiple institutions may have introduced heterogeneity that influenced outcomes. This was a potential factor influencing the outcomes, particularly the operative time and the non‐reproduction of some findings from meta‐analyses. However, our findings may reflect early implementation experience with the robotic platform in Taiwan. Third, comparing our mixed procedural design (right, left, sigmoid colectomy) with studies focusing on single procedure types was challenging. The mixed procedural design may have introduced heterogeneity. However, our study included separate sub‐group analyses for ‘Right Colectomy’ and ‘Combined Left and Sigmoid Colectomy’ to specifically address these differences, which allowed us to identify that the longer operative time and shorter length of stay in the RAC group were driven by the left/sigmoid colectomy subgroup, and the complication benefit was seen only in the right colectomy subgroup. Our mixed procedural design provides broad insights unlike most studies focusing on single colectomy types. Fourth, the full advantages of the robotic platform—such as its utility in extended lymphadenectomy and intracorporeal anastomosis—were not definitively demonstrated due to incomplete data on procedural details. Fifth, this study lacks long‐term oncological outcomes. However, we noted that our short‐term data found comparable local recurrence rates between groups. Finally, information on cost differences between RAC and LAC was unavailable. However, we highlighted that the finding of a shorter length of hospital stay (1 day shorter overall for RAC) is a tangible benefit that could translate to significant benefits for both patients and healthcare systems, including reduced healthcare costs.

In conclusion, this study represents the first multi‐institutional retrospective analysis in Taiwan comparing the clinical outcomes of RAC and LAC. Our real‐world data suggest that RAC is a safe and feasible surgical approach, offering clinical outcomes comparable to LAC and a shorter length of hospital stay. To obtain more definitive and comprehensive results, a prospective study employing a standardized robotic colectomy protocol and incorporating long‐term follow‐up to valuate definitive oncological outcomes (disease‐free and overall survival) is warranted.

## Funding

This work was supported by grants through funding from the National Science and Technology Council (NSTC 112‐2314‐B‐037‐050‐MY3, NSTC 114‐2314‐B‐037‐103‐MY3, NSTC 114‐2321‐B‐037‐003) and the Ministry of Health and Welfare (MOHW113‐TDU‐B‐222‐134014) and funded by the health and welfare surcharge on tobacco products, and the Kaohsiung Medical University Hospital (KMUH113‐3R31, KMUH113‐3R32, KMUH113‐3R33, KMUH113‐3M58, KMUH113‐3M59, KMUH‐S11412, KMUH‐SH11403), Kaohsiung Medical University Research Center Grant (KMU‐TC113A04) and National Tsing Hua University‐Kaohsiung Medical University Joint Research Project (NTHU‐KMU‐KT114P008). In addition, this study was supported by the Grant of Taiwan Precision Medicine Initiative and Taiwan Biobank, Academia Sinica, Taiwan, R.O.C.

## Ethics Statement

This study was approved by the Institutional Review Boards of Kaohsiung Medical University Hospital (KMUHIRB‐E(I)‐20230267), Taichung Veterans General Hospital (CE21319A), Koo Foundation Sun Yat‐Sen Cancer Center (20220608R), Linkou Chang Gung Memorial Hospital (202102412B0), and Shin Kong Wu Ho‐Su Hospital (20220608R).

## Conflicts of Interest

The authors declare no conflicts of interest.

## Data Availability

The data that support the findings of this study are available from the corresponding author upon reasonable request.
